# Assessing racial differences in North American hereditary hemorrhagic telangiectasia study recruitment and care

**DOI:** 10.1186/s13023-025-03883-1

**Published:** 2025-08-28

**Authors:** Gabriella Scott, Ashlee Agundiz, Jeffrey Nelson, Steven Hetts, Marianne Clancy, Helen Kim, Marie E. Faughnan, the Brain Vascular Malformation Consortium HHT Investigator Group

**Affiliations:** 1 Toronto HHT Centre, Keenan Research Centre in the Li Ka Shing Knowledge Institute, St Michael’s Hospital, Toronto, ON Canada; 2 Division of Respirology, Department of Medicine, St. Michael’s Hospital, University of Toronto, Toronto, ON Canada; 3 Department of Anesthesia and Perioperative Care, University of California, UCSF HHT Center of Excellence, San Francisco, San Francisco, CA USA; 4 Department of Radiology, University of California, San Francisco, UCSF HHT Center of Excellence, San Francisco, CA USA; 5 Cure HHT, Monkton, MD USA; 6 St. Michael’s Hospital, 30 Bond St, Toronto, ON M5B1W8 Canada

## Abstract

**Background:**

There is increasing evidence of health outcome disparities due to inequitable healthcare. These inequities are likely compounded in rare disease care and research. We aimed to identify disparities in access to clinical care and research for patients with hereditary hemorrhagic telangiectasia (HHT) in North America.

**Methods:**

We collected race data from the Toronto HHT Centre Brain Vascular Malformation Consortium (BVMC) recruits, the UCSF HHT Centre BVMC recruits, and the UCSF HHT Centre clinic patients, and compared proportions to local populations (2016 Canadian Census and 2010 San Francisco Bay Area Census).

**Results:**

At the UCSF HHT center, there was a significant association between race and BVMC enrollment status (*p* = 0.033). The proportion of White BVMC recruits was significantly higher than reported in the SF Bay Area Census data (*p* < 0.001). At the Toronto HHT centre the proportion of White BVMC recruits was significantly higher than reported in the Canadian Census provincial data (*p* < 0.001), and Toronto Metropolitan Area data (*p* < 0.001).

**Conclusion:**

We report preliminary evidence of racial differences in access to HHT care and research in North America. Our findings indicate a need for race data collection and reporting, as well as identification of barriers and potential solutions in HHT care and research.

## Introduction

Research has increasingly demonstrated inequitable access to healthcare in North America, disproportionately benefiting white people [1–4]. Racial inequity in access to care results from a broad range of barriers, which can be traced back to racism at the structural, institutional, and individual level [5]. Unequal access to medical care results in substantial health disparities among racialized communities. Further, non-inclusive medical research reinforces the exclusion of racialized populations within the medical system, further perpetuating health outcome disparity. Such disparities may be magnified within rare disease care and research specifically [6].

Hereditary Hemorrhagic Telangiectasia (HHT) is a rare autosomal dominant disease [7], affecting approximately 1 in 5,000 people [8]. HHT causes telangiectasias in mucous membranes and skin, as well as vascular malformations in organs such as the liver, lungs, and brain, causing stroke and life-threatening hemorrhage, though preventative therapy is available [7]. HHT is underdiagnosed, and patients often experience delayed diagnosis due to a lack of awareness of the disease [9], and therefore delayed access to treatment. The delayed diagnosis of HHT also impacts care for family members, who are at 50% risk given the autosomal inheritance [7]. It is likely that there are disparities at many stages throughout the course of HHT care and research, further impacting health outcomes in people with this disease.

In an effort to promote equity, diversity, and inclusion (EDI) in Hereditary Hemorrhagic Telangiectasia care, we aimed to identify if there are racial differences between HHT patients recruited for research and HHT patients in the general population. We specifically studied HHT patients recruited to the Brain Vascular Malformation Consortium (BVMC) study at two HHT Centers of Excellence, and compared these to local census populations, as well as to one HHT center’s patient population. Our long-term goal is to develop an approach to further study and address disparities in HHT research and clinical care.

## Materials and methods

### Toronto HHT centre

The Toronto HHT Centre, Canada’s leading HHT centre, consists of an interprofessional team of clinicians, providing specialized care to patients and families with HHT. The centre is in downtown Toronto, Ontario, at a tertiary care hospital, St. Michaels Hospital, with a broad catchment area spanning across the province. The Toronto HHT Centre has not routinely collected race-based data from their clinic population, and therefore race demographics of the Toronto HHT Centre clinic population are not available.

### UCSF HHT center of excellence

The University of California San Francisco (UCSF) HHT Center is one of the leading HHT centers in North America, specialized in providing HHT screening, diagnosis, and care. Located in San Francisco, California the center attracts a diverse patient population. The UCSF HHT Center has routinely collected race data from their clinic population according to the National Institute of Health (NIH) definitions.

### Recruits to the BVMC, HHT project

The Brain Vascular Malformation Consortium (BVMC) (HHT Project) is a multicenter longitudinal observational study with the primary aim research in brain vascular malformations, but also other aspects and outcomes in HHT [10]. The BVMC is part of the Rare Disease Clinical Research Network (RDCRN). When patients are recruited to the BVMC, the data that is collected includes organ involvement, symptomology, and demographics such as race and ethnicity, using NIH definitions.

### Data

Data was collected from Toronto HHT Centre BVMC recruits, UCSF HHT Center BVMC recruits and UCSF HHT Center clinic patients. To account for the broad catchment area, and variation in diversity across the province, the Toronto HHT Center BVMC recruits were divided into participants living within the Toronto Census Metropolitan Area and the broader provincial area. For comparison to local census populations, data was obtained from the 2016 Canadian Census [11] and the 2010 San Francisco Bay Area Census [12].

From the BVMC recruits, gender, ethnicity, race, and city of residence were routinely collected. For BVMC recruits, race and ethnicity were collected using NIH definitions. The race categories included American Indian or Alaska Native, Asian, Black or African American, Native Hawaiian or Other Pacific Islander, White, Refused, Unknown or Not Reported, and/or Other, and ethnicity included Hispanic, Latino, or Spanish origin [13]. During BVMC recruitment, patients were asked to self-identify their race and ethnicity categories. For the purposes of analysis of the BVMC data, we collapsed ethnicity responses into race; we considered patients reporting both Hispanic ethnicity and race of White or Other as Hispanic. Indigenous Canadians recruited to the BVMC were recorded as “American Indian or Alaska Native” on recruitment to the BVMC.

In the Canadian Census racial categories are defined differently than the BVMC dataset. Thus, in order to make comparisons between the two, we collapsed racial categories from the Canadian census data to match the BVMC categories, as feasible. We compared the Asian group from the BVMC to the combined groups of Chinese, Filipino, Japanese, Korean, South Asian, Southeast Asian, and West Asian from Canadian census. We compared the White group from the BVMC to the combined groups of Arab and “Not a visible minority” groups from Canadian census. Indigenous Canadians are reported as a part of the “Not a visible minority” group in the Canadian census, and we compared these to Canadian BVMC recruits recorded as “American Indian or Alaska Native”. In some cases, such as with the BVMC’s “Hispanic” group, there was not a racial category or set of categories in the Canadian census that matched well enough to allow for comparison.

Similar to the BVMC, the American census racial categories are based on the NIH definitions, therefore we did not collapse any of these categories for comparison purposes.

### Statistical analyses

Three different datasets were analyzed for this report.

#### Dataset 1: BVMC participants enrolled at st. Michaels hospital

The first dataset included demographic data of adult HHT patients enrolled in the BVMC at the Toronto HHT Centre, St. Michaels Hospital from 2010 to 2022. We restricted this dataset to patients who were reported to be living in the province at the time of enrollment. We calculated summary statistics for age at study enrollment, gender, and racial and ethnic backgrounds. We calculated the binomial exact 95% confidence interval for the proportion of females in the study and used a one-sample proportion test to determine whether that proportion differed from 0.5. We calculated 95% confidence intervals for the proportions of the racial categories using Sison and Glaz’s [14] method of simultaneous confidence intervals using R’s MultinomialCI package [15]. We compared the proportions of racial categories in the first dataset to the proportions of racial categories in the Ontario province provided by the 2016 Canadian census [11]. When a racial group’s size was sufficiently large in the census data (> 10% of the population, which was the case for the Asian and White groups), we tested whether that proportion differed from the proportion we observed in the BVMC using one-sample proportion tests.

We performed a subgroup analysis of the Toronto HHT Centre BVMC data repeating all the methodology explained above except restricting the analysis to patients who were reported to live within the Toronto metropolitan area (TMA) at the time of analysis. We compared the proportions of racial categories of this subgroup to the proportions of racial categories in the Toronto metropolitan area provided by the 2016 Canadian census.

#### Dataset 2: BVMC participants enrolled at UCSF

The second dataset included demographic data of HHT patients enrolled in the BVMC at the HHT Center of Excellence, University of California, San Francisco (UCSF) from 2011 to 2022. We restricted this dataset to patients who were living in the San Francisco (SF) Bay Area at the time of enrollment. We analyzed this dataset as detailed above for Dataset 1. Using one-sample proportion tests we compared racial proportions from this dataset to the local population census data collected in 2010 [12]. We used a chi-square goodness-of-fit test to determine whether there was an overall difference in the racial makeup of UCSF’s BVMC recruits from the SF Bay Area and the census data. When groups were sufficiently large in the census data (> 10% of the population, which was the case for Asian, Hispanic, and White), we tested whether that proportion differed from the proportion we observed in the BVMC using one-sample proportion tests.

#### Dataset 3: UCSF HHT clinic patients

The third dataset included HHT clinic patients at the HHT Center of Excellence, UCSF. A small proportion of these patients were also enrolled in the BVMC study. We summarized the demographics of the clinic patients and stratified by whether they were enrolled in the BVMC or not. We used a two-sample t-test allowing for unequal variances to test whether the age differed between those enrolled in the BVMC and those who were not enrolled. We calculated the binomial exact 95% confidence interval for the proportion who were female and used a one-sample proportion test to determine whether that proportion differed from 0.5. We used a chi-square test to determine whether females were more or less likely than males to be enrolled in the BVMC after visiting the clinic. We calculated simultaneous 95% confidence intervals for the proportions of the racial categories. We used a chi-square test to determine if racial category was associated with whether a patient was enrolled in the BVMC. The BVMC preferentially enrolls HHT patients with brain arteriovenous malformations (BAVM), aiming for 25% of BVMC enrollment patients to have BAVM. To ensure any identified racial differences between enrolled and unenrolled patients in the chi-square analysis were not an artifact of BAVM status, we performed a likelihood ratio test comparing a two-factor logistic regression model (enrollment = race + BAVM) to a nested model (enrollment = BAVM).

Data analysis was conducted on Stata 15.1 [16], except for the chi-square goodness-of-fit test and when calculating simultaneous confidence intervals for which we used R version 4.2.1 [17]. BVMC data was collected and managed using REDCap electronic data capture tools hosted by the Rare Diseases Clinical Research Network’s Data Managing Coordinating Center at Cincinnati Children’s Hospital [18, 19].

## Results

### BVMC (Toronto HHT Centre) recruits compared to Canadian census data (Tables 1 and 2)

**Table 1:** Population differences between Toronto HHT centre BVMC recruits (Ontario) and Canadian census (Ontario)

Toronto HHT centre recruits from Ontario province (*N* = 369)				Ontario province census data			*P*-value
Racial/Ethnicity	Enrolled	Percent	95% CI	Racial/Ethnicity	Percent	Group Percent	
American Indian or Alaska Native	2	0.5	(0.0–3.6)	Indigenous Canadian	2.8	2.8	DNT
Asian	22	6.0	(3.3–9.1)	Asian *	19.9	19.9	< 0.001
Black or African American	7	1.9	(0.0–5.0)	Black	4.7	4.7	DNT
Hispanic	4	1.1	(0.0–4.2)	Latin American	1.5	1.5	
Native Hawaiian or other Pacific Islander	0	0	(0.0–3.1)				
White	328	88.9	(86.2–92.0)	Not a visible minority	67.9	69.5	< 0.001
				Arab	1.6		
Other	2	0.5	(0.0–3.6)	Visible minority not included elsewhere	0.7	0.7	
Multiple races selected	4	1.1	(0.0–4.2)	Multiple visible minorities	1.0	1.0	

*Includes Chinese, Filipino, Japanese, Korean, South Asian, Southeast Asian, West Asian

95% CI are calculated using Sison and Glaz’s method for simultaneous confidence intervals ^**2,3**^

P-values compare the percent of a recruited racial/ethnic category to the group percent from the census data using a one-sample proportion test; DNT = did not test

**Table 2:** Population differences between Toronto HHT centre BVMC recruits (TMA) and Canadian census (TMA)

Toronto HHT centre BVMC recruits from Toronto Metropolitan Area (*N* = 151)				Toronto Metropolitan Area Census Data			*P*-value
Racial/Ethnicity	Enrolled	Percent	95% CI	Racial/Ethnic	Percent	Group percent	
American Indian or Alaska Native	0	0	(0.0–6.2)	Indigenous Canadian	0.8	0.8	DNT
Asian	20	13.3	(7.3–19.4)	Asian*	36.8	36.8	< 0.001
Black or African American	5	3.3	(0.0–9.5)	Black	7.5	7.5	DNT
Hispanic	2	1.3	(0.0–7.5)	Latin American	2.3	2.3	
Native Hawaiian or other Pacific Islander	0	0	(0.0–6.2)				
White	119	78.8	(72.8–85.0)	Not a visible minority	47.8	49.6	< 0.001
				Arab	1.8		
Other	2	1.3	(0.0–7.5)	Visible minority not included elsewhere	1.3	1.3	
Multiple races selected	3	2.0	(0.0–8.2)	Multiple visible minorities	1.7	1.7	

*Includes Chinese, Filipino, Japanese, Korean, South Asian, Southeast Asian, West Asian

TMA = Toronto Metropolitan Area

95% CI are calculated using Sison and Glaz’s method for simultaneous confidence intervals ^**2,3**^

P-values compare the percent of a recruited racial/ethnic category to the group percent from the census data using a one-sample proportion test; DNT = did not test

A total of 369 adult HHT patients were enrolled in the BVMC at the Toronto HHT Centre and resided within the Ontario province. The median age at study enrollment was 52 with an interquartile range (IQR) of 40–61 and a range of 18–88. Two hundred nineteen (59.4%) were female. Participants were significantly more likely to be female than male (95% CI for proportion of females: 54.1 – 64.4%, *p* < 0.001). Most recruits self-reported as White (*n* = 328, 88.9%, 95% CI: 86.2 – 92.0%). The proportion of White recruits was significantly higher than reported in Canadian Census (Ontario) data (88.9% vs. 69.5%, *p* < 0.001). The second largest category was Asian (*n* = 22, 6.0%, 95% CI: 3.3 – 9.1%), which was significantly lower than the proportion in Canadian Census (Ontario) data (6.0% vs. 19.9%, *p* < 0.001). Two recruits identified as “Indigenous Canadian” (recorded as “American Indian or Alaska Native” in NIH definitions).

A total of 151 adult HHT patients were enrolled in the BVMC at the Toronto HHT Centre and resided within the Toronto Metropolitan Area (151/369, 40% of the Ontario recruits). The median age at study enrollment was 51 with an IQR of 39–60 and a range of 18–88. Eighty-eight (58.3%) were female. Participants were significantly more likely to be female than male (95% CI for proportion of females: 50.4 – 66.1%, *p* < 0.001). Most recruits self-reported as White (*n* = 119, 78.8%, 95% CI: 72.8 – 85.0%). The proportion of White recruits was significantly higher than reported in Canadian Census (Toronto Metropolitan Area) data (78.8% vs. 49.6%, *p* < 0.001). The second largest group was Asian (*n* = 20, 13.3%, 95% CI: 7.3 – 19.4%), which was significantly lower than the proportion in the Canadian Census (Toronto Metropolitan Area) data (13.3% vs. 36.8%, *p* < 0.001).

### BVMC (UCSF HHT Center) recruits compared to SF Bay area census data (Table 3)

**Table 3:** Differences between UCSF HHT center BVMC recruits (SF Bay area) and SF Bay area census

UCSF HHT center BVMC recruits from the San Francisco Bay Area (*N* = 67)				San Francisco Bay Area Census Data	*P*-value
Racial/Ethnicity	Recruited	Percent	95% CI	Percent	
American Indian or Alaska Native	0	0	(0.0–9.7)	0.3	DNT
Asian	4	6.0	(0.0–15.7)	23.0	0.001
Black or African American	0	0	(0.0–9.7)	6.4	DNT
Hispanic	8	11.9	(4.5–21.6)	23.5	0.026
Native Hawaiian or other Pacific Islander	0	0	(0.0–9.7)	0.6	DNT
White	54	80.6	(73.1–90.3)	42.4	< 0.001
Other	0	0	(0.0–9.7)	0.3	DNT
Multiple races selected	1	1.5	(0.0–11.2)	3.5	DNT

95% CI are calculated using Sison and Glaz’s method for simultaneous confidence intervals ^**2,3**^

P-values compare the percent of a recruited racial/ethnic category to the group percent from the census data using a one-sample proportion test; DNT = did not test

A total of 67 HHT patients were enrolled in the BVMC at the UCSF HHT Center and resided within the SF Bay Area. The median age at study enrollment was 47 with an interquartile range (IQR) of 27–60 and a range of 1–83. Forty-five (67.2%) were female. Participants were significantly more likely to be female than male (95% CI for proportion of females: 54.6 – 78.2%, *p* = 0.005). The observed racial proportions of the BVMC recruit population differed significantly from the proportions reported in SF Bay Area Census data (*p* < 0.001, chi-square goodness-of-fit test). Most recruits self-reported as White (*n* = 54, 80.6%, 95% CI: 73.1 – 90.3%). The proportion of White recruits was significantly higher than reported in the SF Bay Area Census data (80.6% vs. 42.4%, *p* < 0.001). The second largest category was Hispanic (*n* = 8, 11.9%, 95% CI: 4.5 – 21.6%), which was significantly lower than the proportion in the SF Bay Area Census data (11.9% vs. 23.5%, *p* = 0.026). The third largest category was Asian (*n* = 4, 6.0%, 95% CI: 0.0 – 15.7%), which was significantly lower than the proportion in the SF Bay Area Census data (6.0% vs. 23.0%, *p* = 0.001).

### UCSF HHT center patients, recruited compared to Non-recruited to the BVMC (Tables 4 and 5)

**Table 4:** UCSF HHT center patient population (*N* = 281)

Racial/Ethnicity	*N*	Percent	95% CI
American Indian or Alaska Native	1	0.4	(0.0–5.9)
Asian	30	10.7	(5.3–16.3)
Black or African American	5	1.8	(0.0–7.4)
Hispanic	46	16.4	(11.0–22.0)
Native Hawaiian or other Pacific Islander	0	0	(0.0–5.6)
White	180	64.1	(58.7–69.6)
Other	11	3.9	(0.0–9.5)
Multiple races selected	8	2.9	(0.0–8.4)

95% CI are calculated using Sison and Glaz’s method for simultaneous confidence intervals ^**2,3**^

**Table 5:** UCSF HHT center patient population: enrollment status is significantly associated with race (*N* = 281, *p* = 0.033)

Racial/Ethnicity	Observed enrolled	Expected enrolled	Observed unenrolled	Expected unenrolled	Chi-square contribution
American Indian or Alaska Native	0	0.3	1 (100%)	0.7	0.4
Asian	3 (10%)	8.4	27 (90%)	21.6	4.9
Black or African American	0	1.4	5 (100%)	3.6	2.0
Hispanic	18 (39%)	12.9	28 (61%)	33.1	2.8
Native Hawaiian or other Pacific Islander	0	0	0	0	0
White	56 (31%)	50.6	124 (69%)	129.4	0.8
Other	1 (9%)	3.1	10 (91%)	7.9	2.0
Multiple races selected	1 (13%)	2.2	7 (88%)	5.8	1.0

Values are counts or count (row percentage)

The overall rate of enrollment was 79/281 (28%)

A total of 281 HHT patients visited UCSF HHT Center and had racial data available. Of those, 79 (28.1%) were eventually enrolled in the BVMC. The median age at first clinic visit was 50 with an IQR of 30–65 and a range of 2–84. There was no significant difference in age between patients enrolled to the BVMC and those who were not (unenrolled) (*p* = 0.727). One-hundred sixty-nine (60.1%) of center patients were female. Center patients were significantly more likely to be female than male (95% CI for females: 54.4 – 65.9%, *p* = 0.001). Additionally, amongst center patients, females were more likely to be enrolled in the BVMC than males (33.1% vs. 20.5%, *p* = 0.021).

Most center patients reported as White (*n* = 180, 64.1%, 95% CI: 58.7 – 69.6%). Hispanic was the second largest reported category (*n* = 46, 16.4%, 95% CI: 11.0 – 22.0%) and Asian was the third (*n* = 30, 10.7%, 95% CI: 5.3 – 16.3%). There was a significant association between race and enrollment status (*p* = 0.033). Table 5 shows the expected and observed numbers of enrolled patients for each race category. The largest contribution to the chi-square test statistic (which demonstrates the association between race and enrollment status) came from the Asian group, which enrolled at a rate that was lower than expected (expected = 8.4, observed = 3). The second largest chi-square contribution was from the Hispanic group, which enrolled at a rate that was higher than expected (expected = 12.9, observed = 18). A likelihood ratio test confirmed that race was a significant factor even when considering BAVM status (*p* = 0.006).

## Discussion

We report preliminary evidence of racial differences between HHT center clinic patients and surrounding populations. We also report an association between race and enrollment to an HHT research study, at an HHT center. This work is preliminary and has its limitations but lays the foundation for beginning to study and addressing disparities in HHT care and research.

Specifically, within the BVMC recruits from the Toronto HHT Centre, we found a significantly higher proportion of White patients and a significantly lower proportion of Asian patients compared to what would be expected from the Canadian census data for Ontario and the Toronto Metropolitan Area. Similarly, within the BVMC recruits from UCSF, we found a significantly higher proportion of White patients, and a significantly lower proportion of Asian and Hispanic patients compared to the SF Bay Area census data. The most important limitation of this study is the comparison to census data, and the assumption that proportion of people with HHT should be similar across racial groups. Although HHT has been reported in most countries [20–22] and races [23], there is limited published data about the prevalence of HHT across races and across regions [24]. Despite this limitation, our study observations suggest that there may be barriers or disparities in how people with HHT in the North American general population can access HHT research. These barriers might exist at many levels from underdiagnosis of HHT in the community, barriers to referral to an HHT centre, barriers to patients’ attending an HHT centre, barriers or disparities in participating in research, and more. In other words, the observed disparities may be due to disparities in clinical care, recruitment to research, or both.

When we compared BVMC study recruits to the clinic population at UCSF HHT Center, where they were recruited, we also observed a significant association between race and enrollment status to the BVMC study. Specifically, the Asian group were enrolled at a lower rate than expected, and the Hispanic group were enrolled at a higher rate than expected. This observation further supports the concern that there are disparities in the recruitment to research.

It should be noted that we observed gender differences. Specifically, we found that participants were significantly more likely to be female than male in each group. While HHT is autosomal dominant, suggesting that it would affect males and females equally, other HHT literature has similarly observed a higher prevalence of women than men [25]. While this was not the primary aim of our study, it is an interesting incidental finding that may point to the need for further research into gender discrepancies in HHT research and care.

There are several limitations that warrant discussion. First, as detailed above, there is minimal published data regarding prevalence by racial group. This is a problem for our study but also for future work addressing barriers and solutions to disparities in HHT care and research. We challenge the HHT scientific community to collect and include detailed racial and other sociodemographic data in scientific publications, so we can begin to resolve this knowledge gap. Secondly, given the rarity of HHT and small patient numbers, we have combined and collapsed racial groups, with the goal of providing preliminary observations, but we recognize that this misrepresents and underrepresents the diversity of the population, and we aim to eventually report more granular data on the HHT population. There are also limitations associated with the variable race and ethnicity definitions amongst our study datasets, and the census data sets, as well as the data collection limitations of the censuses themselves.

Given the considerable differences between the American and Canadian healthcare systems, there is expected variation in the barriers to research and healthcare access between the two countries. As such, theorized causes for our observed racial differences may not be equally applicable to both HHT Centre sites. It should also be noted that our work cannot address the generational effect of post-immigration participation in clinical care at the specialist level and furthermore research participation, this will need to be addressed in future work as concerted efforts are made to include all individuals with this disease. Future research would also benefit from considering the observed differences in the context of other possible causes, beyond health and research access. Finally, we have demonstrated racial differences in access to HHT care and research in this study, but have not identified barriers and their solutions, though it is our long-term goal to do so in partnership with patient communities.

## Conclusions

We report preliminary evidence of racial differences in access to HHT care and HHT research in North America. Our findings indicate a need for race data collection and reporting, as well as identification of barriers to HHT care and research and potential solutions, in partnership with HHT patient communities. Our ultimate goal is to promote equity, diversity, and inclusion in HHT research and care.

## Data Availability

The local population datasets can be access by the public through Statistics Canada (https://www12.statcan.gc.ca/census-recensement/2016/dp-pd/prof/index.cfm?Lang=E) and the Bay Area Census (http://www.bayareacensus.ca.gov/bayarea.htm). Requests to access datasets from the UCSF HHT Center and the BVMC can be made to the corresponding author at marie.faughnan@unityhealth.to.
